# Subtypes and phylogenetic analysis of *Blastocystis* sp. isolates from West Ismailia, Egypt

**DOI:** 10.1038/s41598-022-23360-0

**Published:** 2022-11-09

**Authors:** Shahira A. Ahmed, Heba S. El-Mahallawy, Samar Farag Mohamed, Maria Cristina Angelici, Kyriacos Hasapis, Taisir Saber, Panagiotis Karanis

**Affiliations:** 1 Department of Parasitology, Faculty of Medicine, Suez Canal University, Ismailia, 41522 Egypt; 2 Department of Animal Hygiene, Zoonoses and Animal Behaviour and Management, Faculty of Veterinary Medicine, Suez Canal University, Ismailia, 41522 Egypt; 3 Department of Family Medicine, Faculty of Medicine, Suez Canal University, Ismailia, 41522 Egypt; 4 Department of Environment and Health, Istituto Superiore Di Sanità, Rome, Italy; 5 Department of Basic and Clinical Sciences, University of Nicosia Medical School, 24005, Nicosia, Cyprus; 6 Department of Clinical Laboratory Sciences, College of Applied Medical Sciences, Taif University, P.O. Box 11099, Taif, 21944 Saudi Arabia; 7 Medical Faculty and University Hospital, University of Cologne, Cologne, Germany; 8 Department of Basic and Clinical Sciences, University of Nicosia Medical School, 24005, CY-1700 Nicosia, Cyprus

**Keywords:** Microbiology, Parasitology

## Abstract

In Egypt, *Blastocystis* sp. is not yet on the diagnostic list of parasitology reports, and information about its subtypes (STs) is scarce. This study investigated its prevalence and its STs/alleles, performed phylogenetic analysis, and considered the distribution of risk factors associated with *Blastocystis* sp. infections in West Ismailia, Ismailia governorate. Sociodemographic data, exposure factors, and previous parasitic infection status were recorded for symptomatic and asymptomatic individuals. Microscopy, polymerase chain reaction, sequencing, and phylogenetic analysis for *Blastocystis* sp. isolated from fecal samples were performed. Eighty *Blastocystis* sp.-infected individuals (15.3%) were examined. The age of the individuals ranged between 0.60 and 85.0 (mean 17.10 ± 15.70), the male/female ratio was 33/47, and the asymptomatic/symptomatic ratio was 55/25. The findings demonstrate clear evidence of direct contact with animals, poor water quality, and previous parasitic infections. Eleven samples yielded three *Blastocystis* STs (ST1: allele 4, ST2: alleles 9 and 12, and ST3: allele 34), with ST3 (45.5%) representing the most common subtype. Phylogenetic analysis with a robust bootstrap revealed three distinct clades for isolates of each subtype. This study updates the epidemiological knowledge of the distribution of *Blastocystis* sp. STs in Egypt and expands the current understanding of the prevalence, risk factor frequencies, and genetic diversity of this protist in the studied area.

## Introduction

*Blastocystis* sp. is a unicellular eukaryotic protist in the stramenopile family that lives in the guts of humans and animals. *Blastocystis* sp. has become a much more common issue for public health than previously thought because it is widely distributed with a high incidence and a high degree of genetic diversity^1–3^.

There are about 28 recognized *Blastocystis* sp. lineages. These subtypes (STs) are defined based on the genetic diversity of their small-subunit ribosomal RNA (SSU) genes. By 2013, 17 different STs (ST1 to ST17) had been identified. Although 11 further STs (ST18 to ST28) have been proposed, the validity of four of these (ST18, ST19, ST20, and ST22) remains contested^3,4^. The first nine subtypes and ST12 were isolated from the gastrointestinal tract of humans, with single instances of ST10, ST13, ST14, and ST16 also having been observed^3,5–10^. ST3, ST1, ST2, and ST4 are the most prevalent subtypes in humans. Rodents, birds, pigs, and other primates have all been colonized by different *Blastocystis* sp. subtypes^3,5–8^.

*Blastocystis* sp. is considered a parasite, and scientific consensus classifies it as a commensal and potentially even beneficial resident of the gut^11,12^. The hypothesis that certain strains within subtypes are pathogenic is under investigation^10,13^. Previous studies have speculated that *Blastocystis* sp. interacts with the host’s gut microbiota^14,15^. However, detailed insights remain lacking. Although *Blastocystis* sp. infections are generally asymptomatic in humans, common symptoms include nausea, anorexia, stomach discomfort, flatulence, and acute or chronic diarrhea. Such clinical manifestations have been suggested to result from the proteases and gut-microbiome dysbiosis caused by *Blastocystis* sp. colonization^16^.

*Blastocystis* sp. transmission is not clearly defined, but a human transmission cycle has been proposed^17–19^. Nonetheless, studies of family units in developed and developing countries have indicated that this pathway remains to be conclusively demonstrated^20,21^. Additional sources of infections appear to be contaminated water^22–25^, close contact with animals^1^, and contaminated soil^20^.

A lack of sanitation and clean water means that most Egyptian governorates are probably at high risk of *Blastocystis* infections, with several studies of the country linking *Blastocystis* sp. to urticaria, irritable bowel syndrome, asthma, and iron deficiency anemia, with infections diagnosed in both healthy (asymptomatic) and symptomatic individuals^26–28^.

Still, *Blastocystis* sp. is not listed as a pathogen in Egypt’s parasitological reports. According to a study on the diagnosis of gastrointestinal parasites by primary health care technicians in El-Kassassin, West Ismailia, *Blastocystis* sp. and *Giardia duodenalis* are completely missing from parasitological diagnosis results^29^.

Studies are lacking in Egypt concerning the epidemiology, molecular genetic diversity, and prevalence of *Blastocystis* sp. in carriers and non-carriers. Given the risk factors in Egypt and the current discussion of *Blastocystis* pathogenicity, this parasite should be included in the Egyptian “medical diagnostic radar.” Egypt has 27 governorates, one of which is Ismailia. The West Ismailia governorate has municipal divisions with rural areas, where domestic farm animals and birds share homes with residents. Inadequate hygiene is predicted, given the low socioeconomic status of the area. However, few molecular studies have been conducted to ascertain the incidence and subtype distribution of *Blastocystis* sp. in the governorate. Therefore, the present study aims to focus more closely on the prevalence, risk factor distribution, subtypes, and phylogenetics of *Blastocystis* sp. in a rural Egyptian community.

## Results

**Figure 1:**
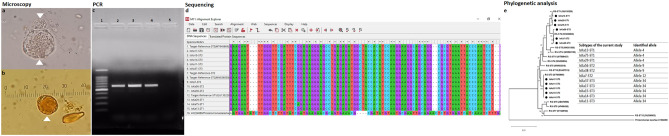
Fecal sample processing steps. (**a**) *Blastocystis* sp. vacuolar form and intermediate phase form using wet-mount examination. (**b**) *Blastocystis* sp. vacuolar form using iodine wet-mount examination. (**c**) PCR of some *Blastocystis*-positive samples. Lane 1 is 100 bp ladder (Promega), lane 2 is the positive control, lanes 3–4 are *Blastocystis*-positive samples, and lane 5 is the negative control. (**d**) Some sequences of *Blastocystis* sp. samples using MEGA software utilizing MUSCLE algorithm. (**e**) The phylogenetic tree of this study isolates’ sequences, cross-correlated with subtype allele.

Microscopy, polymerase chain reaction (PCR), sequencing, and phylogenetic analysis were performed for *Blastocystis* sp. from fecal samples (Fig. 1).

**Figure 2:**
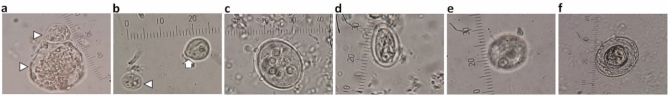
Parasitic stages associated with *Blastocystis* infection. (**a**) *Blastocystis* sp. vacuolar and intermediate-phase (arrowhead) form; (**b**) *Chilomastix mesnili* cyst (arrow) mixed with *Blastocystis* sp. cyst (arrowhead); (**c**) *Entamoeba coli* cyst; (**d**) *Giardia duodenalis* cyst; e: *Entamoeba histolytica*/*dispar*/*moshkovskii* cyst; (**f**) *Hymenolepis nana* egg. Photos a–e were taken with × 100; photo f was taken with × 40.

**Figure 3:**
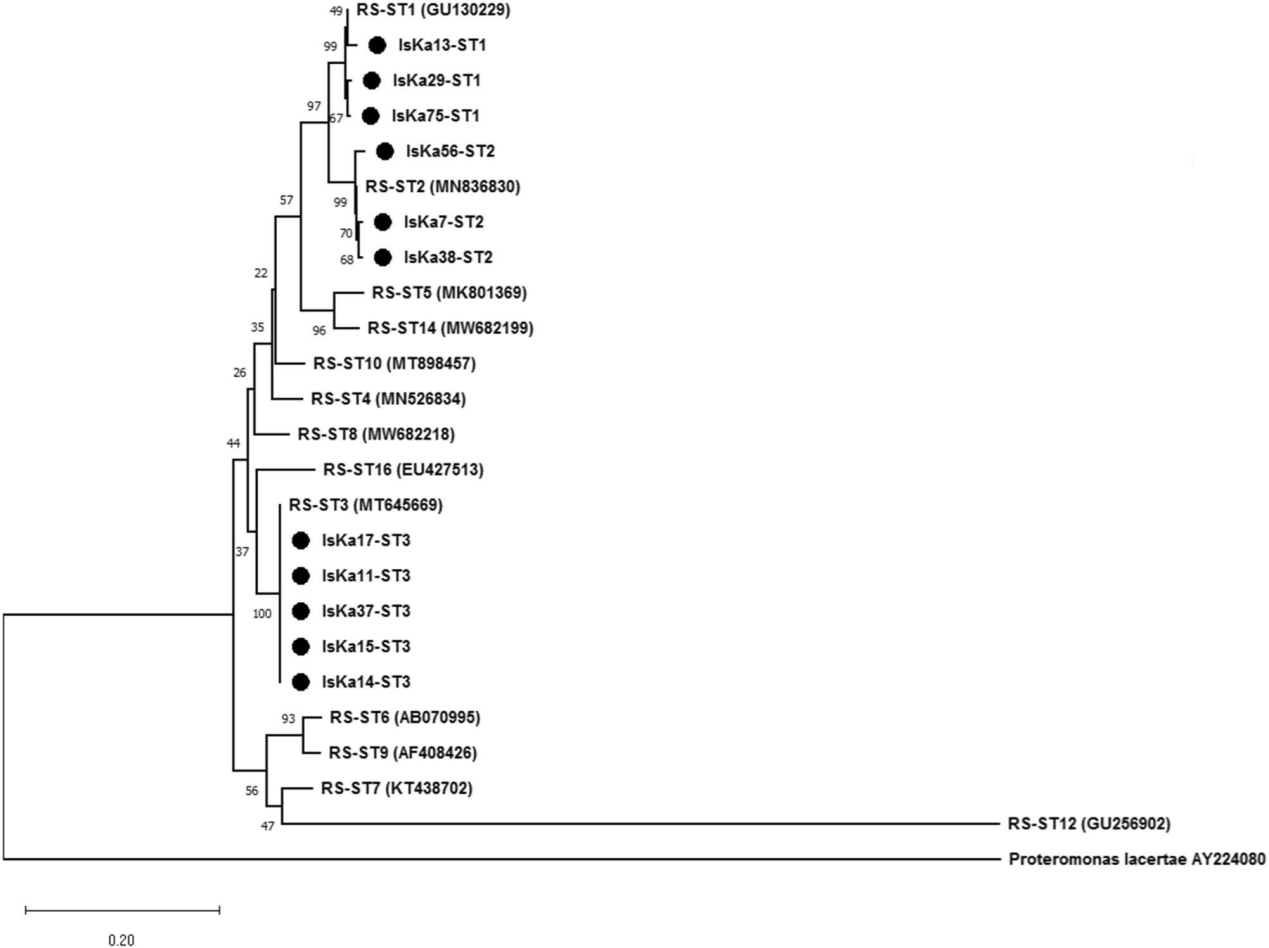
Phylogenetic analysis of the SSU rRNA (small subunit ribosomal RNA) nucleotide sequences of three subtypes of *Blastocystis* isolated from the West Ismailia population. The numbers above the branches indicate the percentages of bootstrap samplings. A solid circle indicates the *Blastocystis* species isolated in the current study, with *Proteomonas lacerate* used as an outgroup. RS: Reference sequence; IsKa: Ismailia–Kassassin isolates of the current study.

Descriptive data concerning patient sociodemographics and the distribution of risk factors is illustrated for the 80 *Blastocystis-*positive samples (Tables 1 and 2). Microscopic and molecular examinations of *Blastocystis-*positive samples are shown in detail (Table 3, Fig. 2, and Table 4). Of the 15 samples sent for sequencing, 11 were successfully sequenced with high-quality yield (see "Methods" section for details). The genetic diversity and genotype distribution of *Blastocystis* sp. isolates are detailed in Table 5. Information regarding *Blastocystis* sp. STs in the current investigation, compared against studies of different Egyptian governorates, appears in Table 6. The current study’s sequence isolates were submitted to phylogenetic analysis (Fig. 3).

**Table 1:** Descriptive data concerning the sociodemographic and risk factors of *Blastocystis-*positive subjects in West Ismailia, Egypt.

Subjects participated in the study	Category	Item	Category	*Blastocystis* sp.–positive subjects No. (%)
SAD & NAD (n = 80)	Sociodemographic data	Gender	Male	33 (41.3)
			Female	47 (58.8)
		Age in years	< 5	7 (8.8)
			5–18	52 (65)
			> 18–30	10 (12.5)
			> 30	11 (13.8)
			Min.–Max.	0.60–85.0
			Mean ± SD	17.10 ± 15.70
			Median (IQR)	12.0 (6.0–19.0)
SAD & NAD (n = 80)	Symptomatology status	Clinical symptoms	Symptomatic	25 (31.3)
			Asymptomatic	55 (68.75)
		Related symptoms in symptomatic patients	Abdominal pain	11 (13.8)
			Fever	2 (2.5)
			Abdominal pain & fever	1 (1.3)
			Vomiting & abdominal pain	2 (2.5)
			Vomiting, fever & abdominal pain	1 (1.3)
			Diarrhea & abdominal pain	5 (6.3)
			Diarrhea, fever & abdominal pain	1 (1.3)
			Diarrhea & vomiting	1 (1.3)
			Diarrhea, vomiting, fever, abdominal pain dehydration	1 (1.3)
SAD (n = 33)	Exposure factors	Have source of water	Yes	32 (96.7)
			No	1 (3)
		Type of water source to human	Tap without filter	26 (78.8)
			Pump	1 (3)
			Buying containers	6 (18.2)
		Water quality	Clear	26 (32.5)
			Yellow	1 (3)
			Turbid	6 (18.2)
		Have domestic animals	Yes	30 (90.9)
			No	3 (9.1)
		Direct contact with animals	Poultry	6 (18.2)
			Poultry & livestock	8 (24.2)
			Poultry & pets	9 (27.4)
			All types of animals	7 (21.2)
			No animals	3 (9.1)
		Type of animal farm ground (n = 30)	Cement	7 (23.3)
			Sand	23 (76.6)
		Direct contact with animal (n = 30)	Yes	23 (76.6)
			No	7 (23.3)
		Type of water source to animal (n = 30)	Tap	21 (70)
			Canal	2 (6.7)
			Pump	7 (23.3)

SAD: Subjects with available data, *n* = 33; NAD: Subjects with no available data, *n* = 47 (“These individuals were recruited through primary health care units during Ministry of Health and Population programs to eradicate schistosomiasis, [and] only gender, age, and symptoms were available”); IQR: Interquartile range; SD: Standard deviation; sp.: species.

**Table 2:** Previous parasitic infection and its association with anti-parasitic treatment in *Blastocystis-*positive subjects in West Ismailia, Egypt.

PPI for SAD (n = 33)	Treatment		X^2^	^MC^P
	Yes (n = 14)	No (n = 19)		
*E.* complex	2 (14.3%)	1 (5.3%)	8.232	0.029*
*E. coli*	1 (7.1%)	0 (0%)		
*H. nana*	9 (64.3%)	6 (31.6%)		
*Schistosoma* sp.	2 (14.3%)	10 (52.6%)		
No previous parasitic infection	0 (0%)	2 (10.5%)		

PPI: Previous parasitic infections within the last year; SAD: Subjects with available data; *E.* complex: *Entamoeba histolytica*/*dispar*/*moshkovskii*; *E. coli*: *Entamoeba coli*; *H. nana*: *Hymenolepis nana*; sp.: Species; *χ^2^ Chi-squared test, MC: Monte Carlo *P*: *p* value for comparing between the studied groups, *statistically significant at *p* ≤ 0.05.

**Table 3:** Results of microscopic examination for *Blastocystis-*positive isolates in West Ismailia (*n* = 80).

Microscopic examination results	No. (%)	Comment
*Blastocystis* sp.	47 (58.8)	Single infection (47) Double infection (27) Triple infection (5) Quadruple infection (1)
*Blastocystis* sp. & *E. coli*	19 (23.8)	
*Blastocystis* sp. & *E*. complex	5 (6.3)	
*Blastocystis* sp. & *G. duodenalis*	2 (2.5)	
*Blastocystis* sp. & *C. mesnili*	1 (1.3)	
*Blastocystis* sp., *E. coli* & *C. mesnili*	3 (3.8)	
*Blastocystis* sp., *E. coli* & *G. duodenalis*	1 (1.3)	
*Blastocystis* sp., *E. coli* & *H. nana*	1 (1.3)	
*Blastocystis* sp., *E. coli*, *G. duodenalis*, & *H. nana*	1 (1.3)	

Sp.: Species; *E. coli*: *Entamoeba coli*; *E*. complex: *Entamoeba histolytica*/*dispar*/*moshkovskii*; *C. mesnili*: *Chilomastix mesnili*; *G. duodenalis*: *Giardia duodenalis*; *H. nana*: *Hymenolepis nana.*

**Table 4:** Molecular subtyping of the sequenced isolates (*n* = 11).

*Subtypes	No. (%)	Comment
ST1	3 (27.3)	ST3 was the commonest subtype
ST2	3 (27.3)	
ST3	5 (45.5)	

*15/80 isolates were sent to Rome, Italy, for sequencing, with only 11/80 sequences yielding high-quality sequence results.

**Table 5:** The distribution of *Blastocystis* sp. genotypes and genetic diversity among the 11 sequenced isolates from West Ismailia.

No	Patient code	Sex/Age in years	Symptomatic (1)/Asymptomatic (2)	Mixed infection with other parasites	Subtype of *Blastocystis* sp.	No. of Nucleotide differences	GenBank Accession No. of this study isolates	Subtype Alleles
1	IsKa -7	M/15	2	*E. coli*	ST -2	3	OL845605	Allele 12
2	IsKa -11	F/23	2	–	ST -3	–	OL845603	Allele 34
3	IsKa -13	M/28	2	*E. coli*	ST -1	5	OL845610	Allele 4
4	IsKa -14	M/6	2	–	ST -3	–	OL845602	Allele 34
5	IsKa -15	M/9	2	*E. coli, C. mesnili*	ST -3	–	OL845601	Allele 34
6	IsKa -17	M/10	2	–	ST -3	–	OL845600	Allele 34
7	IsKa -29	M/28	2	–	ST -1	4	OL845608	Allele 4
8	IsKa -37	F/17	2	–	ST -3	–	OL845604	Allele 34
9	IsKa -38	F/45	2	–	ST -2	3	OL845607	Allele 9
10	IsKa -56	M/8	2	–	ST -2	5	OL845606	Allele 9
11	IsKa -75	F/6	1	–	ST -1	3	OL845609	Allele 4

IsKa: Ismailia–Kassassin; *E. coli*: *Entamoeba coli*; *C. mesnili*: *Chilomastix mesnili*. M: male; F: Female.

**Table 6:** Molecular genotyping data of *Blastocystis* sp. in the Egyptian population from several governorates.

Country/Region	Group of study and associated comorbidity	Associated GIT symptoms	^2^Molecular methods	Species/STs	Most prevalent subtype/allele	Reference
Egypt/Ismailia	West Ismailia subjects	Asymptomatic Symptomatic	PCR Sequencing	*Blastocystis* sp./ ST1, ST2 and ST3	ST3/ Allele no. 34	The current study
Egypt/Beni-Suef	Patients with colorectal cancer and non-cancer individuals	NR	PCR Sequencing	*Blastocystis* sp./ ST1, ST2, ST3 and ST7	ST3	^41^
Egypt/Cairo	Patients with urticaria unresponsive to urticarial TTT	Asymptomatic ^1^Symptomatic	PCR RFLP	*Blastocystis* sp./ ST3	ST3	^26^
Egypt/Beni-Suef	Patients with IBS	Symptomatic	PCR Sequencing	*Blastocystis* sp./ ST3 & ST1	ST3	^27^
Egypt/Cairo	Anaemic and non-anaemic individuals	NR	PCR	*Blastocystis* sp.*/ *ST1 & ST3	ST1	^28^
Egypt/Cairo	Symptomatic patients	Diarrhoea, abdominal pain	nPCR Sequencing	*Blastocystis* sp./ ST1, ST2 & ST3	ST3	^50^
Egypt/Ismailia	Symptomatic and asymptomatic individuals	Asymptomatic ^1^Symptomatic	STS primers	*Blastocystis* sp./ ST1, ST2 & ST3	ST3	^51^
Egypt/Mansoura	Patients with IBS	Asymptomatic ^1^Symptomatic	PCR–RFLP	*Blastocystis* sp./ ST1, ST2, ST3 & ST4	ST3	^32^
Egypt/Cairo	Asthmatic and non-asthmatic patients	Asymptomatic Symptomatic (urticaria + IBS)	PCR–RFLP	*Blastocystis* sp./ ST3 & ST4	ST3	^33^
Egypt/Alexandria	Symptomatic and asymptomatic individuals	Asymptomatic ^1^Symptomatic	PCR–RFLP	*Blastocystis* sp./ ST1, ST2, ST3 & ST4	ST3	^52^
Egypt/Cairo	Patients with IBS and healthy individuals	Asymptomatic Symptomatic	STS primers	*Blastocystis* sp./ ST1, ST2, ST3 & ST4	ST3	^53^
Egypt/Ismailia	Symptomatic and asymptomatic individuals	Asymptomatic ^1^Symptomatic	STS primers	*Blastocystis* sp./ ST1, ST2, ST3 & ST4	ST3	^54^
Egypt/Cairo	Patients with urticaria and healthy individuals	Asymptomatic Symptomatic	PCR–RFLP	*Blastocystis* sp./ ST3	ST3	^30^
Egypt/Ismailia	Human with DCA and domestic animals	Asymptomatic	STS primers	*Blastocystis* sp./ ST1, ST2, ST3 & ST4	ST3	^37^
Egypt/Kafr-Elsheikh	Human with DCA and human with no DCA	Asymptomatic Symptomatic	PCR Sequencing	*Blastocystis* sp./ ST1, ST2 & ST3	ST2	^39^

^1^Symptomatic cases have the following symptoms as a single presentation or in combination: diarrhea, abdominal pain, vomiting, flatulence, fatigue, and fever; ^2^The target gene in all studies was small subunit rRNA; STS: sequence-tagged site primers; IBS: Irritable bowel syndrome; RFLP: Restriction fragment-length polymorphism; nPCR: nested polymerase chain reaction; NR: Not reported; sp.: Species; DCA: Direct contact with animals.

*Blastocystis* sp. was identified in a total of 80/520 (15.3%) fecal samples, according to direct light microscopy of smears (saline and iodine examination). The 80 *Blastocystis*-positive subjects included those with no available data (NAD = 47) and those with complete historical data available (SAD = 33). For NAD samples, the only available data were age, sex, and symptomatology status, with no information on exposure factors or previous parasitic infections (PPIs). Both SAD and NAD subjects were classified as “symptomatic” (gastrointestinal symptoms) or “asymptomatic” (no gastrointestinal symptoms), based on responses to a standardized questionnaire (Table 1).

The 80 *Blastocystis*-positive individuals ranged in age from 0.6 to 85 years. Most patients (55/80) had no gastrointestinal symptoms; those who did mostly complained of abdominal pain and diarrhea. Other non-specific symptoms such as vomiting, dehydration, and fever were also described (Table 1). Those aged between 5 and 18 (52/80) were most affected by *Blastocystis* sp. infection (Table 1). Approximately 60% (31) of this group were asymptomatic; the remainder were symptomatic. Females in this group represented 55.8% (29), and males represented 44.2% (23).

Most of the SAD subjects reported owning domestic animals (poultry, livestock, and pets). About 76.6% reported direct contact with their animals and that the floors of their animal farms were covered with sand. The water supplies at the studied subjects’ residences appeared mostly clear. However, some participants mentioned that the water sometimes became yellow and turbid with a smell (Table 1).

Almost all SAD individuals––31/33 (93.9%)––had recorded previous parasitic infections (PPI), including *Entamoeba* sp., *Schistosoma* sp., and *Hymenolepis nana*, in the previous year. Only 14 patients had received the anti-parasitic treatment they needed, with schistosomiasis patients, in particular, mostly not taking their medications or following their prescribed treatment regimens (Table 2).

According to microscopic wet-mount examinations of their fecal samples, 41.3% of participants recorded mixed parasitic infections (pathogenic and non-pathogenic). Polyparasitism included infections that were doubled, tripled, or even quadrupled. Other protozoan and helminthic parasites were found in *Blastocystis*-infected subjects, including *Entamoeba coli*, *Entamoeba histolytica*/*dispar*/*moshkovskii*, *Chilomastix mesnili*, *Giardia duodenalis*, and *Hymenolepis nana* (Table 3, Fig. 2).

Only 11 samples were successfully amplified, producing high-quality sequences that identified three STs (ST1, ST2, and ST3). *Blastocystis* ST3 was the most common ST in the West Ismailia population (45.5%), followed by ST2 and ST1 (27.3% each) (Table 4).

Tests performed using BLAST software revealed that each of the SSU rRNA gene sequences obtained in this study demonstrated a high degree of homology with previously reported sequences from other *Blastocystis* sp. isolates. Although ST1 and ST2 sequences showed some single-nucleotide polymorphism mutations against the reference strains, ST3 demonstrated 100% homology with the reference strain. Furthermore, 10/11 sequenced isolates belonged to asymptomatic subjects, with only one isolate (ST1) belonging to a symptomatic subject (Table 5).

The alignment of *Blastocystis* sequences showed three to five nucleotide differences. Three samples––IsKa 7 (ST2), IsKa 38 (ST2), and IsKa 75 (ST1)––demonstrated three nucleotide differences. Despite these differences, the BLAST search revealed that the first two of these isolates belong to the same *Blastocystis* ST. One sample, IsKa 29 (ST1), showed four nucleotide differences, while two samples, IsKa 13 (ST1) and IsKa 56 (ST2), showed five nucleotide differences. All of the sequence data for *Blastocystis* nucleotides obtained from the current study were deposited in GenBank with accession numbers OL845600–OL845610 as follows: ST3 represented by five samples (OL845600–OL845604), ST2 represented by three samples (OL845605–OL845607), and ST1 represented by three samples (OL845608–OL845610) (Table 5). In terms of *Blastocystis* alleles, ST3 and ST1 produced completely homogeneous isolates with alleles 34 and allele 4, respectively. Meanwhile, ST2 exhibited low genetic variation in one isolate (IsKa 7), which corresponded to allele 12, with its remaining two isolates corresponding to allele 9.

A phylogenetic tree was constructed using eleven nucleotide sequences representative of the current study and GenBank database reference sequences, with *Proteromonas lacertae* (U37108) used as the outgroup. Three subtypes were distinguishable: ST1, ST2, and ST3 (Fig. 1).

## Discussion

This study’s findings revealed *Blastocystis* sp. to be prevalent in 15.3% of the West Ismailia population, which is consistent with the observations of El-Badry et al. (2018). Other studies have revealed variations in *Blastocystis* prevalence^27,30,31^. PCR in Cairo revealed a prevalence of 35.5% for *Blastocystis* in both patient and control groups^30^. Patients with irritable bowel syndrome in Beni-Suef were found to have a prevalence of *Blastocystis* of 16.5% by microscopy and 19.1% by culture^27^*.* In the same governorate, 53.6% of patients with acute diarrhoea had *Blastocystis*^31^. Such disparities can be attributed to various epidemiological parameters, including the target population, detection method, and the presence or absence of symptoms.

In this study, *Blastocystis* colonization was more common among children aged 6 to 18 than among children under five years old and adults, which aligns with two previous Egyptian studies observing schoolchildren to be the most affected^32,33^. Infection with *Blastocystis* sp. has also been demonstrated by global surveys to be prevalent among schoolchildren^18,34–36^, likely owing to this age group’s lax hygiene standards. *Blastocystis* infection age disparities may be influenced by associated exposure risk factors, children’s immunity, and environmental variables^34–36^*.*

This study classifies *Blastocystis* infections as silent, with most infected subjects not exhibiting symptoms. Three factors may influence the asymptomatic status of *Blastocystis* infections among Egyptians: (a) awareness, which is limited in the rural Egyptian population, of the need to seek medical attention for diarrhea; (b) the diagnostic ability of Egyptian laboratories to identify *Blastocystis* sp. in primary care settings (i.e., primary health care units), leaving *Blastocystis* outside their diagnostic scope^29^; and (c) in the case of *Blastocystis* infection, the concept that it is a commensal protist, which encourages Egyptian physicians to disregard treating patients despite the presence of distressing symptoms^29^. These factors may enable *Blastocystis* sp. to colonize the host for an extended period without causing disease.

Most Egyptians, and particularly those living in rural areas, keep domestic animals and birds in and around their homes. This is especially evident in West Ismailia, where domestic animals, especially poultry, coexist with humans in traditional Egyptian residences without separate yards, exposing owners to high concentrations of *Blastocystis* infective stages for extended periods. This has led to the prediction that the risk of *Blastocystis* infection is tenfold greater in rural areas than in urban areas among the Egyptian population with irritable bowel syndrome^27^. Elsewhere, strong molecular evidence has confirmed zoonotic transmission between animals and their human caregivers^37,38^. Notably, humans in direct contact with animals were found to have the same *Blastocystis* STs in two studies conducted in Northern and Eastern Egypt (ST1, ST2, ST3, and ST4)^37,39^. Additionally, poultry can harbor the human-transmissible *Blastocystis* ST6 and ST7^40^, although this has only been reported in a recent Egyptian study of colorectal cancer patients^41^.

The Sweet Water Canal serves as the primary water source for municipal divisions in West Ismailia and is used directly for animal bathing, dishwashing, and laundry. Consequently, water used for residential or recreational purposes becomes contaminated, creating a risk of transmission of gastrointestinal diseases to humans and animals alike. Rural communities in West Ismailia have been particularly hard-hit, with some settlements in remote locations lacking access to safe drinking water, which increases the possibility of contamination during transportation and processing. Numerous protozoan contaminants, including *Blastocystis* sp., have been detected in Egypt’s Dakahlia, Ismailia, and El-Minia governorates in potable water, water tanks, pumps, waterworks, and surface water (i.e., River Nile, ponds, and canals)^24,42,43^. Most SAD residents were observed to utilize tap water without a filter, with the water supplied appearing clear. However, because it is only used to remove rust, insects, and dust, the presence or absence of a filter would not substantially impact the purification of water from protozoa stages. Thus, even if the water appears clear, it may contain *Blastocystis* sp. Furthermore, in recent years, soil pollution has been identified as a source of *Blastocystis* sp. infection^20^; as a major farming region, soil is extremely likely to be a source of infection for the West Ismailia population.

PPI are prevalent in rural Ismailia (author’s observation, unpublished data); when questioned, this study’s participants were fully aware of this. Over half of participants failed to take their parasitic infection medications as prescribed and did not complete the entire course of treatment (dose and duration). Consequently, infected individuals have acted as carriers facilitating anthroponotic protist transmission, while also experiencing infection maintenance, chronicity, and consequences.

This study’s investigations also revealed polyparasitism in the West Ismailia population. Although most samples represented single *Blastocystosis* infections, the presence of pathogenic (*G. duodenalis*, *E. histolytica*, *H. nana*) and non-pathogenic (*E. coli*, *C. mesnilli*, non-pathogenic species of *E. histolytica*) parasites mixed with *Blastocystis* sp. infection suggests multiple sources of infection. Mixed parasitic infections are highly predicted in rural areas due to the presence of multiple risk factors, as has been previously documented^44–47^. Consumption of contaminated water and unwashed vegetables, lack of fingernail trimming and hand washing, children playing in the dirt, barefoot walking, low socioeconomic status, lack of sanitation, and large numbers of family members sharing a single room have all been observed among the residents of West Ismailia^27,31,48,49^. Furthermore, rats, cockroaches, fleas, ants, and flies were observed to spread in numerous locations, especially during hot weather, alongside sewage rash. Such behavioral, social, and sanitary factors are almost certainly implicated in developing mixed parasitic infections and perpetuating the life cycle of those infections. Thus, nearly all the transmission routes required for *Blastocystis* sp. appear open.

Three subtypes of *Blastocystis* sp. were characterized in the current investigation via the molecular analysis of isolates, namely, ST3, ST2, and ST1, with the latter two recording equal distribution. This study’s findings corroborate those of Souppart et al. (2010), who discovered that ST3 had the highest prevalence (61.9%) and that ST1 and ST2 had equal prevalence (19.05%).

Several Egyptian studies have subtyped and sequenced *Blastocystis* sp.^39,41,50^, with five STs (ST1, ST2, ST3, ST4 and ST7) identified at varying frequencies in distinct Egyptian groups using PCR sequenced-tagged sites, PCR restriction fragment length polymorphisms, and PCR sequencing (barcoding) (Table 6). The high prevalence of *Blastocystis* sp. ST1–ST4 in the Egyptian community suggests that most infections are transmitted from person to person.

The current investigation has revealed that ST3 is responsible for the vast majority of *Blastocystis* infections in West Ismailia, aligning with observations for other Egyptian governorates across 12 other studies (Table 6), which have revealed ST3 to be the most prevalent *Blastocystis* subtype in six distinct Egyptian locations; furthermore, it is the ST most closely related to various gastrointestinal symptoms (Table 6). Other subtypes (ST1, ST2, ST4 and ST7) have been detected in the Egyptian community, with varying frequencies depending on the sample size and testing technique used. ST1 and ST2 have been identified as relevant STs in a smaller number of Egyptian studies^28,39^. Notably, STs and their relative frequencies appear to vary significantly within a single country (Table 6).

Almost all the isolates sequenced in this investigation were asymptomatic, except for one patient who suffered diarrhea and abdominal pain and was subtyped as ST1, an observation consistent with the findings of other studies^10,55–57^. *Blastocystis* is more frequent in healthy individuals, with its existence also linked to altered composition and increased richness of the bacterial gut microbiota^14,15^. It is unclear whether *Blastocystis* directly promotes a healthy gut and microbiome or whether it prefers to colonize and persist in a healthy gut environment. A study of *Blastocystis* sp. ST3 indicated that *Blastocystis* sp. may modify the gut ecosystem in a protective manner and facilitate faster recovery from disturbances^12^. The presence of *Blastocystis* among healthy individuals has also been linked to reduced levels of fecal calprotectin, a sign of intestinal inflammation according to a comparative investigation conducted in Mexico^56^. In contrast, some researchers have suggested that particular *Blastocystis* sp. isolates may produce an imbalance of the gut microbiota^58–63^. The context of the environment and hosts must be considered when discussing whether *Blastocystis* is a pathogen or a mutualist.

The present study’s genetic analysis reveals that all 11 isolates detected in *Blastocystis* subtypes cross-corresponded to previously observed alleles. Intriguingly, ST3 isolates produced the highest frequency of isolates matching allele 34, the most common variant found in humans worldwide, with ST2 isolates exhibiting low levels of genetic diversity and multiple nucleotide substitutions corresponding to two different alleles (9 and 12). Although ST1 isolates demonstrated limited genetic diversity, all isolates corresponded to allele 4. In Egyptian isolates from Cairo, genetic diversity was detected in three subtypes in the same pattern, with ST1 and ST2 exhibiting nucleotide differences ranging from 1 to 11 and ST3 exhibiting reduced genetic variability of up to four nucleotide differences^50^. However, no further allelic analysis was conducted. On the contrary, there was no evidence of genetic diversity in the *Blastocystis* subtypes (ST3 and ST1) isolated from individuals with irritable bowel syndrome in the Beni-Suef governorate^27^.

A phylogenetic tree demonstrated that the 11 nucleotide sequences in this study clustered into the same subtype cluster, with high bootstrap support, and could be classified into three subtypes: ST1, ST2, and ST3. Each *Blastocystis* sp. ST formed a distinct clade, implying that the West Ismailian *Blastocystis* population can be divided into three subgroups. Another Egyptian study found the same phylogenetic distribution pattern for ST1 and ST3 subtype clusters in patients with irritable bowel syndrome^27^.

Among the drawbacks of the present investigation is the infeasibility of sequencing every isolate. Because samples were collected via a large-scale survey, there were no epidemiological data for some participants, which hindered presenting comprehensive information.

*Blastocystis* sp. infections are significantly under- and mis-diagnosed in Egypt, particularly in rural and remote areas such as West Ismailia. Additional research can illuminate the epidemiological situation of *Blastocystis* sp. in Egypt, enabling more effective control efforts against *Blastocystis* sp. infections and other parasitic disorders.

## Conclusions

The current study updates the epidemiological situation and distribution of *Blastocystis* STs in Egypt. Phylogenetic analysis has revealed three distinct clades for isolates pertaining to each subtype, adding to our current understanding of *Blastocystis*’s prevalence and genetic diversity. The widespread presence of ST3 in the West Ismailia population and throughout Egypt necessitates subtyping analysis, which has become indispensable for elucidating the relationship between *Blastocystis* subtypes and pathogenicity in the Egyptian population.

We highlight the need to invest in parasite education programs, specific to *Blastocystis* sp., that involve the general public along with doctors and laboratory technicians. Moreover, further studies are needed in the underrepresented areas of Egypt to verify the distribution of *Blastocystis* sp. throughout the country.

## Methods

### Study area and sample collection

The Ministry of Health and Population in West Ismailia’s municipal divisions (villages around the Sweet Water Canal’s geographical line) conducted a screening survey of 598 fecal samples for schistosomiasis eradication campaigns. Samples were collected randomly, without regard for age or gender, from the nearest sampling area.

**Figure 4:**
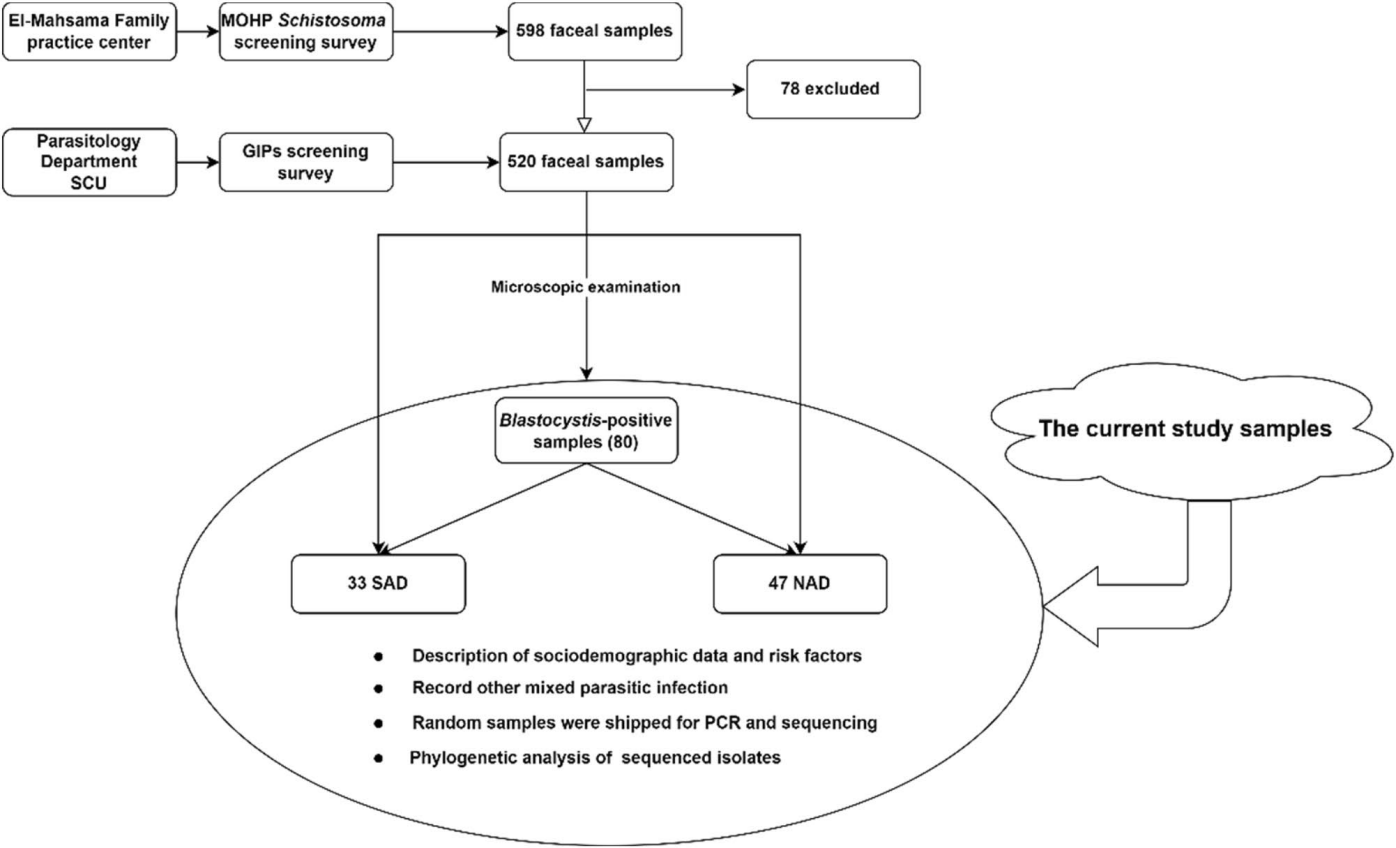
Flowchart describing the origin of the current study’s samples. SAD: Subjects with available data; NAD: Subjects with no available data; MOHP: Ministry of Health and Population; GIPs: Gastrointestinal parasites; SCU: Suez Canal University.

Thus, this descriptive study used data from screens for gastrointestinal parasites of individuals in the municipal divisions of West Ismailia (El-Kassassin, El-Mahsama, El-Talelkbeer, and Abu-Suwayr). A total of 80 fecal samples positive for *Blastocystis* sp. were analyzed. The flowchart in Fig. 4 describes the current study’s process.

Five hundred and twenty samples were selected after considering their suitability based on inclusion and exclusion criteria. The 520 fecal samples and their questionnaire forms were sent to Suez Canal University’s Parasitology Laboratory for ovum and parasite examination, using wet mount and iodine microscopy to screen for gastrointestinal parasites. *Blastocystis*-positive samples were separated and selected for the current study analysis. The amount of the received fecal samples (a full tablespoon, i.e., 15–20 gr) was used as a guideline for rolling in or rolling out the received fecal samples.

To collect and transfer the fecal samples, participants were given a clean, labeled plastic container with an applicator stick. Patients were given verbal explanations in Arabic regarding the collection and transfer processes and the amount of stool sample required. Stool samples were excluded if contaminated with urine and water or if the amount was too small (less than a full tablespoon, i.e., 15–20 gr). Subsequently, the parasitology laboratory at El-Mahsama Family Practice Center divided the stool samples into two parts: one part for the eradication program campaign (primary health care unit at the parasitology laboratory) and one part for processing at the Suez Canal University’s Medical Department’s parasitology laboratory in Ismailia.

### Microscopy of fecal samples

A teaspoon-sized fecal sample was combined with 50 mL saline. In a 15 mL conical plastic tube, the mixture was strained using gauze. One mL of the filtered fecal mixture was pipetted into Eppendorf tubes and frozen. For *Blastocystis* form diagnosis, microscopic examination was conducted using a direct smear. To view other parasitic stages under the microscope, the formalin-ethyl acetate sedimentation procedure was employed to concentrate the strained mixture^64^. Wet-mount analysis was performed and Lugol’s iodine added to the slides for microscopic examination.

### Extraction of genomic DNA

Before extraction, the preserved 1 mL fecal samples were washed and centrifuged several times in phosphate-buffered saline until the supernatant became clear. Then, the supernatant was discarded, and 200 µL of the 1 mL sediment was exposed to the InhibitEX lysis buffer from the Qiagen DNA Stool Mini Kit (Qiagen, Germany, GmbH) according to the manufacturer’s protocol. Using 100 µL of elution buffer, the protocol was slightly modified, and the extracted DNA was stored at -20 °C for further molecular investigation.

### PCR amplification and sequencing

Financial restrictions meant that only 15 samples were sent to the Istituto Superiore di Sanità in Rome, Italy, for molecular characterization. For amplification of the SSU rRNA region in *Blastocystis* isolates, Blasto F (5′-TCTGGTTGATCCTGCCAGT-3′) and Blasto R (5′-AGCTTTTTAACTGCAACAACG-3′) primers were used according to the protocol described by Meloni et al.^65^. A proofreading enzyme (GoTaq Promega) was used to amplify the PCR. The Promega Wizard SV Gel and PCR Clean-Up System kit was used to purify the obtained 600 bp amplicon, and purified products were sent for MGW Sanger sequencing.

The sequences obtained were compared with other *Blastocystis* SSU rRNA gene sequences available in the National Center for Biotechnology Information database using the BLAST tool (http://www.ncbi.nlm.nih.gov). Multiple nucleotide sequence alignments were performed using the MUSCLE algorithm (https://www.megasoftware.net/web_help_10/Part_II_Assembling_Data_For_Analysis/Building_Sequence_Alignments/MUSCLE/About_Muscle.htm) of the MEGA software (https://www.megasoftware.net/)^3,66^. Additionally, *Blastocystis* alleles were identified by determining their closest similarity to known *Blastocystis* sequences using the *Blastocystis* PubMLST database (https://pubmlst.org/organisms/blastocystis-spp).

### Phylogenetic analysis

Molecular and Evolution Genetic Analysis software (https://www.megasoftware.net/) was used to produce a phylogenetic tree for nucleotide sequences using the maximum likelihood method. One thousand bootstrap replicates were used to test the phylogenetic tree’s reliability and the statistical support for the topology. Evolutionary distances were calculated using the Tamura-3 parameter model.

### Statistical analysis

A descriptive analysis was used to report sociodemographic characteristics and the frequency and distribution of *Blastocystis* subtypes among the affected subjects. After uploading all questionnaire data, the IBM SPSS software package version 26.0 was used to analyze the data (IBM Corporation, Armonk, NY). Numbers and percentages were used to represent categorical data; mean and standard deviation were used to represent numerical data. The chi-squared test was used to examine the relationship between a PPI and its treatment as two qualitative variables. The *p*-value was statistically significant at 0.05.

### Ethical considerations

Fecal sample collection and medical history questionnaires were reviewed and approved by the Scientific Research Committee and Bioethics Board of Suez Canal University, Faculty of Medicine, Egypt (approval no. 5089). All methods were performed in accordance with the relevant guidelines. Participants in the study were asked to sign a written informed consent form that clearly detailed in Arabic the study’s objectives, sociodemographic questionnaire, symptomatology, exposure factors, and procedures. The collected data were kept private during and after the collection and analysis. The study’s participants were informed that they could withdraw at any time. Patients whose fecal specimens were positive for gastrointestinal parasites were referred to physicians for further treatment.

## Data Availability

All data generated or analyzed during this study are included in this published article.

## References

[1] Ahmed, S. A. & Karanis, P. *Blastocystis* spp., ubiquitous parasite of human, animals and environment. in *Reference Module in Earth Systems and Environmental Sciences* 1–6 (Elsevier, 2019). 10.1016/B978-0-12-409548-9.10947-9.

[2] Scanlan PD (2014). The microbial eukaryote *Blastocystis* is a prevalent and diverse member of the healthy human gut microbiota. FEMS Microbiol. Ecol..

[3] Stensvold C, Clark CG (2020). Pre-empting pandora’s box: *Blastocystis* subtypes revisited. Trends Parasitol..

[4] Hublin JSY, Maloney JG, Santin M (2021). *Blastocystis* in domesticated and wild mammals and birds. Res. Vet. Sci..

[5] Maloney JG, da Cunha MJR, Molokin A, Cury MC, Santin M (2021). Next-generation sequencing reveals wide genetic diversity of *Blastocystis* subtypes in chickens including potentially zoonotic subtypes. Parasitol. Res..

[6] Maloney, J. G., Jang, Y., Molokin, A., George, N. S. & Santin, M. wide genetic diversity of *Blastocystis* in white-tailed deer (Odocoileus virginianus) from Maryland, USA. *Microorganisms* **9**, (2021). 10.3390/microorganisms9061343 PMC8233720 34205799

[7] Maloney, J. G., Molokin, A., da Cunha, M. J. R., Cury, M. C. & Santin, M. *Blastocystis* subtype distribution in domestic and captive wild bird species from Brazil using next generation amplicon sequencing. *Parasite Epidemiol. Control* **9**, (2020). 10.1016/j.parepi.2020.e00138 PMC6995250 32021915

[8] Higuera A (2021). Identification of multiple *Blastocystis* subtypes in domestic animals from Colombia using amplicon-based next generation sequencing. Front. Vet. Sci..

[9] Ramírez JD (2016). Geographic distribution of human *Blastocystis* subtypes in South America. Infect. Genet. Evol..

[10] Tito RY (2019). Population-level analysis of *Blastocystis* subtype prevalence and variation in the human gut microbiota. Gut.

[11] Mülayim S (2021). investigation of isolated *Blastocystis* subtypes from cancer patients in Turkey. Acta Parasitol..

[12] Billy, V. *et al. Blastocystis* colonization alters the gut microbiome and, in some cases, promotes faster recovery from induced colitis. *Front. Microbiol.* **12**, (2021). 10.3389/fmicb.2021.641483 PMC8058373 33897648

[13] Betts EL (2021). Metabolic fluctuations in the human stool obtained from *Blastocystis* carriers and non-carriers. Metabolites.

[14] Andersen, L. O. brie. & Stensvold, C. R. *Blastocystis* in health and disease: Are we moving from a clinical to a public health perspective? *J. Clin. Microbiol.* **54**, 528 (2016). 10.1128/JCM.02520-15 PMC4767957 26677249

[15] Stensvold CR (2020). Differentiation of *Blastocystis* and parasitic archamoebids encountered in untreated wastewater samples by amplicon-based next-generation sequencing. Parasite Epidemiol. Control.

[16] Mirjalali, H. *et al.* Distribution and phylogenetic analysis of *Blastocystis* sp. subtypes isolated from IBD patients and healthy individuals in Iran. *Eur. J. Clin. Microbiol. Infect. Dis.* **36**, 2335–2342 (2017). 10.1007/s10096-017-3065-x 28741097

[17] Paulos S (2018). Occurrence and subtype distribution of *Blastocystis* sp. in humans, dogs and cats sharing household in northern Spain and assessment of zoonotic transmission risk. Zoonoses Public Health.

[18] Muadica AS (2020). Molecular diversity of *Giardia duodenalis*, *Cryptosporidium* spp. and *Blastocystis *sp in asymptomatic school children in Leganés Madrid Spain. Microorganisms.

[19] Khaled S (2021). *Blastocystis* sp. prevalence and subtypes distribution amongst Syrian refugee communities living in North Lebanon. Microorganisms.

[20] Jinatham V, Maxamhud S, Popluechai S, Tsaousis AD, Gentekaki E (2021). *Blastocystis* one health approach in a rural community of Northern Thailand: Prevalence, subtypes and novel transmission routes. Front. Microbiol..

[21] Lhotská Z (2020). A study on the prevalence and subtype diversity of the intestinal protist *Blastocystis* sp in a gut-healthy human population in the Czech Republic. Front. Cell. Infect. Microbiol..

[22] Taamasri P (2000). Transmission of intestinal blastocystosis related to the quality of drinking water - PubMed. Southeast Asian J Trop Med Public Heal..

[23] Leelayoova S (2004). Evidence of waterborne transmission of *Blastocystis hominis*–PubMed. Am. J. Trop. Med. Hyg..

[24] Elshazly A, Elsheikha H, Soltan D, Mohammad K, Morsy T (2007). Protozoal pollution of surface water sources in Dakahlia Governorate, Egypt–PubMed. J. Egypt. Soc. Parasitol..

[25] Angelici MC, Nardis C, Scarpelli R, Ade P (2018). *Blastocystis hominis* transmission by non-potable water: A case report in Italy–PubMed. New Microbiol..

[26] Abdel-Hameed DMA, Hassanin OM, Zuel-Fakkar NM (2011). Association of *Blastocystis*
*hominis* genetic subtypes with urticaria. Parasitol. Res..

[27] El-Badry AA, Abd El Wahab WM, Hamdy DA, Aboud A (2018). *Blastocystis* subtypes isolated from irritable bowel syndrome patients and co-infection with *Helicobacter pylori*. Parasitol. Res..

[28] Deeb El HK, Khodeer S (2013). *Blastocystis* spp: Frequency and subtype distribution in iron deficiency anemic versus non-anemic subjects from Egypt. J. Parasitol..

[29] Ahmed, S. A., Mohamed, S. F., Fouad, A. M. & Karanis, P. Gastrointestinal parasites diagnoses at the primary health care units: A comparative analysis of diagnostic abilities of parasitology staff technicians versus medical parasitologists in Ismailia, Egypt. *R. Soc. Trop. Med. Hyg.* trac072 (2022). 10.1093/trstmh/trac072 35906091

[30] Zuel-Fakkar NM, Abdel Hameed DM, Hassanin OM (2011). Study of *Blastocystis*
*hominis* isolates in urticaria: A case-control study. Clin. Exp. Dermatol..

[31] Hamdy DA, Abd El Wahab WM, Senosy SA, Mabrouk AG (2020). *Blastocystis* spp. and *Giardia intestinalis* co-infection profile in children suffering from acute diarrhea. J. Parasit. Dis..

[32] Abu El-Fetouh N, Abdelmegeed E, Attia R, El-Dosoky I, Azab M (2015). Genotyping of *Blastocystis hominis* symptomatic isolates and kinetics of associated local CD3 and CD20 cell infiltrate. Parasitol. United J..

[33] El Saftawy EA, Amin NM, Hamed DH, Elkazazz A, Adel S (2019). The hidden impact of different *Blastocystis* genotypes on C-3 and IgE serum levels: A matter of debate in asthmatic Egyptian children. J. Parasit. Dis..

[34] Bertozzo, T. V., David, É. B., Oliveira-Arbex, A. P., Victória, C. & Guimarães, S. Frequency, spatial distribution, and genetic diversity of *Blastocystis* among referred individuals to a clinical laboratory: First report of subtype 9 in Brazil. *Acta. Trop.* **234**, (2022). 10.1016/j.actatropica.2022.106608 35841954

[35] Khaled S (2020). Prevalence and subtype distribution of *Blastocystis* sp. in Senegalese school children. Microorganisms.

[36] Cinek, O. *et al. Blastocystis* in the faeces of children from six distant countries: Prevalence, quantity, subtypes and the relation to the gut bacteriome. *Parasit. Vectors* **14,** (2021). 10.1186/s13071-021-04859-3 PMC8359624 34384477

[37] Mokhtar A, Youssef A (2018). Subtype analysis of *Blastocystis* spp isolated from domestic mammals and poultry and its relation to transmission to their incontact humans in Ismailia governorate, Egypt. Parasitol. United J..

[38] Udonsom R (2018). *Blastocystis* infection and subtype distribution in humans, cattle, goats, and pigs in central and western Thailand. Infect. Genet. Evol..

[39] Abdo SM (2021). Detection and molecular identification of *Blastocystis* isolates from humans and cattle in northern Egypt. J. Parasit. Dis..

[40] Rauff-Adedotun AA, Mohd Zain SN, Farah Haziqah MT (2020). Current status of *Blastocystis* sp. in animals from Southeast Asia: A review. Parasitol. Res..

[41] Ali SH (2022). an association between *Blastocystis* subtypes and colorectal cancer patients: A significant different profile from non-cancer individuals. Acta Parasitol..

[42] Rayan HZ, Eida OM, El-hamshary EM, Ahmed SA (2009). Detection of human *Cryptosporidium* species in surface water sources in Ismailia using polymerase chain reaction. Parasitol. United J..

[43] Khalifa RMA, Ahmad AK, Abdel-Hafeez EH, Mosllem FA (2014). Present status of protozoan pathogens causing water-borne disease in Northern part of El-Minia governorate Egypt. J. Egypt. Soc. Parasitol..

[44] Fernández-Niño JA (2017). Profiles of intestinal polyparasitism in a community of the Colombian Amazon region. Biomedica.

[45] Pagheh AS (2018). A cross-sectional analysis of intestinal parasitic infections among the general population in north of Iran. J. Infect. Dev. Ctries..

[46] Dawaki S, Al-Mekhlafi HM, Ithoi I (2019). The burden and epidemiology of polyparasitism among rural communities in Kano State Nigeria. Trans. R. Soc. Trop. Med. Hyg..

[47] Weerakoon, K. G. *et al.* Co-parasitism of intestinal protozoa and *Schistosoma japonicum* in a rural community in the Philippines. *Infect. Dis. Poverty* **7**, (2018). 10.1186/s40249-018-0504-6 PMC6287361 30526666

[48] Elmonir W (2021). Prevalence of intestinal parasitic infections and their associated risk factors among preschool and school children in Egypt. PLoS ONE.

[49] Yones D, Zaghlol K, Abdallah A, Galal L (2015). Effect of enteric parasitic infection on serum trace elements and nutritional status in upper Egyptian children. Trop. Parasitol..

[50] Souppart L (2010). Subtype analysis of *Blastocystis* isolates from symptomatic patients in Egypt. Parasitol. Res..

[51] Abaza S, Rayan H, Soliman R, Nemr N, Mokhtar A (2014). Subtype analysis of *Blastocystis* spp. isolates from symptomatic and asymptomatic patients in Suez Canal University Hospitals, Ismailia. Egypt. Parasitol. United J..

[52] El-Taweel H (2020). Restriction fragment length polymorphism RFLP analysis of *Blastocystis* spp. in symptomatic and asymptomatic individuals from Alexandria. Egypt. Parasitol. United J..

[53] Fouad SA, Basyoni MMA, Fahmy RA, Kobaisi MH (2011). The pathogenic role of different *Blastocystis hominis* genotypes isolated from patients with irritable bowel syndrome. Arab J. Gastroentrol..

[54] Hussein EM, Hussein AM, Eida MM, Atwa MM (2008). Pathophysiological variability of different genotypes of human *Blastocystis hominis* Egyptian isolates in experimentally infected rats. Parasitol. Res..

[55] Mardani Kataki M, Tavalla M, Beiromvand M (2019). Higher prevalence of *Blastocystis hominis* in healthy individuals than patients with gastrointestinal symptoms from Ahvaz, southwestern Iran. Comp. Immunol. Microbiol. Infect. Dis..

[56] Nieves-Ramírez, M. E. *et al.* Asymptomatic intestinal colonization with protist *Blastocystis* is strongly associated with distinct microbiome ecological patterns. *mSystems* **3**, e00007-18 (2018). 10.1128/mSystems.00007-18 PMC6020473 29963639

[57] Kesuma, Y., Firmansyah, A., Bardosono, S., Sari, I. P. & Kurniawan, A. *Blastocystis* ST-1 is associated with irritable bowel syndrome-diarrhoea (IBS-D) in Indonesian adolescences. *Parasite Epidemiol. Control* **6**, (2019). 10.1016/j.parepi.2019.e00112 PMC6742775 31528737

[58] Yason JA, Liang YR, Png CW, Zhang Y, Tan KSW (2019). Interactions between a pathogenic *Blastocystis* subtype and gut microbiota: In vitro and in vivo studies. Microbiome.

[59] Stensvold CR, Arendrup MC, Nielsen HV, Bada A, Thorsen S (2008). Symptomatic infection with *Blastocystis* sp. subtype 8 successfully treated with trimethoprim-sulfamethoxazole. Ann. Trop. Med. Parasitol..

[60] Stensvold CR, Lebbad M, Verweij JJ (2011). The impact of genetic diversity in protozoa on molecular diagnostics. Trends Parasitol..

[61] Mattiucci S, Crisafi B, Gabrielli S, Paoletti M, Cancrini G (2016). Molecular epidemiology and genetic diversity of *Blastocystis* infection in humans in Italy. Epidemiol. Infect..

[62] Gabrielli, S. *et al.* Molecular Subtyping of *Blastocystis* sp. isolated from farmed animals in Southern Italy. *Microorganisms* **9**, (2021). 10.3390/microorganisms9081656 PMC8399531 34442735

[63] Stensvold CR, Alfellani M, Clark CG (2012). Levels of genetic diversity vary dramatically between *Blastocystis* subtypes. Infect. Genet. Evol..

[64] Cociancic P, Rinaldi L, Zonta ML, Navone GT (2018). Formalin-ethyl acetate concentration, FLOTAC pellet and anal swab techniques for the diagnosis of intestinal parasites. Parasitol. Res..

[65] Meloni D (2011). Molecular subtyping of *Blastocystis* sp. isolates from symptomatic patients in Italy. Parasitol. Res..

[66] Stensvold CR (2007). Terminology for *Blastocystis* subtypes–a consensus. Trends Parasitol..

